# An observational study of socioeconomic and clinical gradients among diabetes patients hospitalized for avoidable causes: evidence of underlying health disparities in China?

**DOI:** 10.1186/1475-9276-13-9

**Published:** 2014-01-30

**Authors:** Brian Chen, Karen Eggleston, Hong Li, Nilay Shah, Jian Wang

**Affiliations:** 1 Arnold School of Public Health, University of South Carolina, 800 Sumter St, Columbia, SC 29208, USA; 2 Walter A. Shorenstein Asia Pacific Research Center, Stanford University, 616 Serra St., Encina Hall E311, Stanford, CA 94305, USA; 3 Department of Endocrinology, Sir Run Run Shaw Hospital, School of Medicine, Zhejiang University, 3 East Qingchun Road, Hangzhou, Zhejiang 310016, China; 4 Division of Health Care Policy and Research, Mayo Clinic, 200 First St. SW, Rochester, MN 55905, USA; 5 Center for Health Policy and Management, Shandong University, Jinan, Shandong 250012, China

**Keywords:** Diabetes, China, Equity, Social gradient, Preventable hospitalizations, Ambulatory care sensitive conditions

## Abstract

**Introduction:**

Diabetes is an ambulatory care sensitive condition that can generally be managed in outpatient settings with little or no need for inpatient care. As a preliminary step to investigate whether health disparities can be detected in the inpatient setting in China, we study how diabetic patients hospitalized without prior primary care contact or with greater severity of illness differ from other diabetic inpatients along socioeconomic and clinical dimensions.

**Methods:**

We conduct an observational study using three years of clinical data for more than 1,800 adult patients with diabetes at two tertiary hospitals in East China. Univariate analysis and probit regression are used to characterize the differences in socioeconomic and clinical factors between patients hospitalized for diabetes with no prior primary care contact and those hospitalized with previous treatment experience. Secondarily, we use ordinary least squares regression to estimate the socioeconomic and clinical differences associated with poor serum glucose control at admission.

**Results:**

We find that compared with patients hospitalized after prior treatment experience, inpatients with no previous primary care contact for diabetes have worse clinical laboratory values, are more likely to be young and male, to have lower education attainment, and to have poorer blood sugar control. Insurance, urban residence, and previous use of diabetic medication are in turn negatively correlated with HbA1c levels upon admission.

**Conclusion:**

Among hospitalized diabetic patients, socioeconomic factors such as lower education attainment, rural residence and lack of full insurance are associated with avoidable hospitalizations or worse indicators of health. Although we cannot definitively rule out selection bias, these findings are consistent with health disparities observable even at the inpatient level. Future studies should study the underlying mechanism by which traditionally vulnerable groups are more likely to be hospitalized for avoidable causes and with greater severity of illness.

## Introduction

In recent years, the burden of chronic illnesses has overtaken that of infectious diseases globally [1]. China, the world’s second largest economy that is home to nearly 20% of the global population [2], represents an important country to study the burden, and in particular inequalities in the burden of chronic illnesses such as diabetes. As in India and Brazil, the prevalence of diabetes in China is predicted to grow at a rate double that seen in the United States [3,4]. A recent study estimates the annual economic burden of diabetes in China at 17.6 billion Chinese Yuan (CNY), or US$ 2.5 billion [5], a burden that can only be expected to worsen as the number of people living with diabetes rises.

Diabetes is currently the eighth leading cause of mortality worldwide [6], and contributes significantly to premature mortality in low and middle-income countries [7]. Yet it is also considered an ambulatory care sensitive condition (ACSCs) that can often be effectively managed in a primary care setting. Inpatient admissions for these conditions are often classified as “preventable hospitalizations” and are considered to be indicative of suboptimal primary care [8]. Examining the factors associated with hospitalizations for ACSCs such as diabetes can help policymakers identify vulnerable populations and formulate appropriate measures to reduce the economic, physical, and emotional burden of preventable hospitalizations.

There is a growing literature on health and socioeconomic status in China [9,10] and around the world [11-13]. Studying the prevalence of diabetes in China, Chan et al. [14] and Yang et al. [4] find that a low level of education is a risk factor for diabetes. Yang et al. [4] also document an association between pre-diabetes and education below the college level. The relationship between insurance status and access to primary care has also been investigated, documenting an association between insurance and improved access among socially disadvantaged groups such as the less educated and the poor [15]. Nevertheless, we are not aware of any previous study that documents a socioeconomic gradient among diabetic *inpatients* in China.

Our primary objective is to investigate, among diabetic inpatients in two Chinese tertiary care hospitals, how diabetic patients hospitalized without prior primary care contact or with greater severity of illness differ from other diabetic inpatients based on observable socioeconomic and clinical characteristics. These differences may point to underlying health disparities in China. First, we investigate the associations between socioeconomic/clinical factors and patient’s prior primary care treatment history for diabetes. We feel that hospitalization almost immediately upon a diagnosis of diabetes (with no other previous contact with the primary care system for the treatment of diabetes) is a particularly troubling first contact with the healthcare system for treatment of an ACSC, which is a medical condition for which timely and appropriate ambulatory care should prevent the need for hospitalizations. Under optimal conditions of access to primary care, DM should ideally have been diagnosed and treated early in the outpatient setting without the need for hospitalization upon diagnosis. As articulated in the consensus statement of the American Diabetes Association and the European Association for the Study of Diabetes, “except in rare circumstances . . . hospitalization is not required for the initiation or adjustment of therapy” [16].

Second, we investigate the socioeconomic factors that are associated with hospitalizations characterized by poorer control of serum glucose, as measured by HbA1c scores (glycated hemoglobin, or blood sugar levels). Tight control of blood sugar levels in diabetic patients is the primary goal of timely and effective primary care and management, so identifying patient groups with poor blood sugar control may help policymakers direct scarce healthcare resources to patients most vulnerable to this category of preventable hospitalizations. Our two research questions together ask whether there is evidence consistent with social gradients existing even at the inpatient level, with traditionally vulnerable patient groups – such as those with low education attainment, rural residence, or incomplete insurance coverage – experiencing more avoidable hospitalizations or greater severity of illness at admission.

## Data and methods

### Data

Our data set includes socioeconomic and clinical information for all adult patients (aged 18 or older) hospitalized with a primary diagnosis of type 2 Diabetes Mellitus (DM) (all inpatients with an ICD 9 CM code of 250.x) (1) between May 1, 2005, and April 30, 2008, at Sir Run Run Shaw Hospital, affiliated with the Zhejiang University Medical School (hereafter SRRSH) (N = 960) and (2) all adults admitted with type 2 DM between 2005 and 2008 at Shandong Provincial Hospital (SPH) located in Jinan, Shandong Province (N = 911).

Both SRRSH and SPH are tertiary care hospitals categorized as Class 3A level providers, the highest level of accreditation for hospitals in China. Table 1 provides the summary statistics of the characteristics of all patients hospitalized for diabetes-related conditions between 2005 and 2008 at the respective hospitals. Because China has never imposed gatekeeping requirements for access to care at any of its hospitals, patients are free to self-refer to providers of any accreditation level regardless of the severity of their illness. There is no theoretical catchment area or predefined population from which SRRSH and SPH draw their patients. In practice, however, these two urban tertiary care hospitals serve a patient population defined by their surrounding urban and rural areas. The primary constraint is that health insurance programs generally provide only minimal coverage for care received at out-of-locality hospitals, but patients willing to pay high out-of-pocket expenses do travel from nearby provinces to seek care at Class 3A hospitals such as SRRSH and SPH.

**Table 1:** Descriptive statistics

			**Reasons for inpatient admission**				**Test of difference in mean†**	
	**All observations**		**Newly diagnosed**		**Treatment-experienced**			
**Variable**	**Mean**	**Std dev**	**Mean**	**Std dev**	**Mean**	**Std dev**	**Diff or ratio**	**95% CI**
**Panel A: Sir Run Run Shaw Hospital**								
	(N = 960)		(N = 134)		(N = 849)			
Patient age	55.60	12.89	49.91	12.18	56.52	12.78	-6.61	[-8.93 - -4.29]
*Female*	0.43		0.28		0.46		0.60	[0.45 - 0.80]
*< High School*	0.58		0.61		0.57		1.07	[0.92 - 1.24]
*Insured*	0.20		0.19		0.21		0.91	[0.62 - 1.32]
*Semi-insured*	0.44		0.42		0.44		0.95	[0.77 - 1.17]
*Uninsured*	0.36		0.40		0.35		1.12	[0.89 - 1.41]
*Urban*	0.44		0.44		0.45		0.99	[0.80 - 1.21]
*County/town*	0.18		0.15		0.18		0.83	[0.54 - 1.27]
*Village*	0.38		0.41		0.37		1.10	[0.88 - 1.37]
*Married*	0.97		0.97		0.97		1.00	[0.97 - 1.04]
*Current smoker*	0.26		0.34		0.24		1.41	[1.08 - 1.84]
Duration of DM (months)	67.30	64.42	4.69	9.69	77.47	63.78	-72.78	[-83.61 - -61.94]
BMI (kg/m^2^)	23.90	4.00	24.78	5.26	23.76	3.74	1.02	[0.27 - 1.77]
Fast. blood sugar (mg/dl)	163.95	66.28	186.60	70.91	160.30	64.90	26.30	[13.88 - 38.72]
Triglyceride mg/dl	201.88	209.51	242.83	265.11	195.37	198.67	47.46	[8.88 - 86.03]
Total cholesterol mg/dl	175.18	44.80	186.16	47.25	173.43	44.17	12.73	[4.50 - 20.96]
Plasma LDL-C mg/dl	103.59	39.81	109.19	33.72	102.72	40.62	6.47	[-1.00 - 13.95]
AST IU/L	27.79	26.05	37.66	48.13	26.21	20.02	11.45	[6.71 - 16.19]
ALT IU/L	30.52	30.51	40.87	36.31	28.86	29.16	12.01	[6.45 - 17.58]
Creatinine mg/dl	0.87	0.55	0.79	0.21	0.88	0.58	-0.10	[-0.20 - 0.003]
BUN mg/dl	15.87	7.77	14.55	5.15	16.08	8.10	-1.53	[-2.96 - -0.11]
eGFR ml/min/1.73 M2	101.39	34.54	111.05	31.22	99.84	34.81	11.22	[4.90 - 17.53]
HbA1c %	8.70	2.82	10.40	2.22	8.41	2.81	1.99	[1.48 - 2.50]
*Complications upon diagnosis*	0.32		0.41		0.30		1.35	[1.07 - 1.69]
*Chronic complications*	0.83		0.75		0.84		0.89	[0.80 - 0.98]
UKPDS CHD risk	24.63	0.69	18.77	1.39	25.59	20.97	-6.82	[-10.66 - -2.97]
UKPDS stroke risk	15.00	0.68	4.34	0.54	16.69	0.77	-12.35	[-16.16 - -8.55]
Drug expenditures	3,100.82	4,588.62	2336.80	2770.30	3231.51	4810.51	-894.71	[-1735.13 - -54.30]
Diagnostic test expenditures	2,106.74	1,131.80	1976.92	873.45	2132.77	1163.81	-155.85	[-362.73 - 51.02]
Surgical expenditures	96.57	456.74	57.44	287.07	103.12	478.82	-45.67	[-129.50 - 38.16]
Hospital room expenditures	693.40	1,039.34	622.01	931.19	706.59	1056.48	-84.58	[-275.30 - 106.14]
Therapy expenditures	309.89	1,625.24	176.82	359.85	332.10	1746.37	-155.28	[-453.59 - 143.04]
Medical material expenditures	596.51	2,297.50	344.97	386.96	638.50	2471.13	-293.53	[-715.04 - 127.98]
Total expenditures	6,903.93	8,328.53	5473.80	3956.79	7135.94	8815.95	-1,662.14	[-3181.44 - -142.84]
**Panel B: Shandong Provincial Hospital**								
	(N = 911)		(N = 62)		(N = 849)			
Patient age	61.61	12.32	52.76	15.47	62.25	11.81	-9.49	[-12.62 - -6.37]
*Female*	0.50		0.44		0.50		0.88	[0.66 - 1.17]
*< High School*	*Variable not available for Shandong data*							
*Insured*	0.09		0.06		0.10		0.60	[0.22 - 1.64]
*Semi-insured*	0.69		0.44		0.71		0.62	[0.47 - 0.82]
*Uninsured*	0.21		0.50		0.19		2.63	[1.98 - 3.50]
*Urban*	0.88		0.71		0.89		0.80	[0.68 - 0.94]
*Rural*	0.12		0.29		0.11		2.64	[1.71 - 4.07]
*Married*	0.94		0.98		0.93		1.05	[1.01 - 1.10]
*Current smoker*	0.18		0.16		0.19		0.84	[0.47 - 1.51]
Duration of DM (months)	120.19	84.49	3.14	2.27	128.74	81.15	-125.60	[-145.83 - -105.36]
BMI (kg/m^2^)	25.30	3.98	25.47	4.66	25.29	3.92	0.18	[-0.93 - 1.30]
Fasting blood glucose (mg/dl)	157.28	44.21	159.69	38.80	157.10	44.60	2.59	[-7.52 - 12.70]
Plasma triglyceride mg/dl	155.04	133.11	166.06	158.28	154.22	131.13	11.84	[-28.53 - 52.21]
Plasma total cholesterol mg/dl	202.32	49.85	205.76	37.06	202.06	50.68	3.70	[-6.13 - 13.54]
Plasma LDL-C mg/dl	133.72	377.89	122.95	27.70	134.52	391.72	-11.57	[-38.81 - 15.67]
AST IU/L	23.79	26.50	30.09	39.57	23.33	25.26	6.76	[-3.24 - 16.76]
ALT IU/L	22.53	33.10	30.03	43.38	21.99	32.19	8.04	[-2.97 - 19.05]
Creatinine mg/dl	1.01	0.59	0.86	0.19	1.02	0.61	-0.16	[-0.22 - -0.10]
BUN mg/dl	17.44	12.29	14.41	5.81	17.67	12.62	-3.26	[-4.94 - -1.58]
eGFR ml/min/1.73 M^2^	*Variable not available for Shandong Data*							
HbA1c %	10.05	2.58	11.03	2.93	9.98	2.54	1.05	[0.38 - 1.71]
*Complication upon diagnosis*	*Variable not available for Shandong data*							
*Chronic complications*	0.90		0.68		0.91		0.75	[0.63 - 0.89]
UKPDS CHD risk	*Variable not available for Shandong data*							
UKPDS stroke risk	*Variable not available for Shandong data*							
Drug expenditures	12,043.97	9,701.05	7,330.60	4,718.68	12,388.17	9,881.62	-5,057.57	[-7541.92 - -2573.21]
Diagnostic test expenditures	1,431.07	1,165.98	1,431.32	1,256.91	1,430.26	1,159.81	1.06	[-300.13 - 302.26]
Surgical expenditures	32.00	235.56	33.87	266.70	31.86	233.30	2.01	[-58.84 - 62.86]
Hospital room expenditures	935.89	1,642.43	574.35	356.56	962.29	1,695.71	-387.94	[-811.48 - 35.60]
Therapy expenditures	2,587.25	3,869.05	1,926.57	1,115.99	2,635.50	3,992.51	-708.93	[-1707.36 - 289.49]
Medical material expenditures	*Variable not available for Shandong data*							
Total expenditures	17,294.27	13,118.62	11,478.48	5,945.20	17,718.98	13,397.19	-6,240.50	[-9605.00 - -2876.00]

^†^Test of difference in means between the sample of patients hospitalized after diagnosis with no primary care (“newly diagnosed”), compared to patients with hospitalized after having had primary care treatment for diabetes. *T*-test performed for continuous variables; prevalence ratios given for categorical variables.

Data: Inpatient admissions for Type 2 Diabetes Mellitus at (1) Sir Run Run Shaw Hospital (Hangzhou, China) (Panel A) and (2) Shandong Provincial Hospital (Jinan, China), 2005–2008.

Observations of patients under 18 are excluded.

We exclude Type 1 Diabetes based on clinical manifestations and progression of the disease, poor islet B cell function, and the presence of Glutamic Acid Decarboxylase autoantibody, insulin auto-antibody, or islet cell antibody. From hospital administrative records, we also code whether patients have health insurance or partial insurance.

Medical records indicate patient self-report of duration of diagnosis. We refer to inpatients who report no previous contact with the primary care system for the treatment of DM as “newly diagnosed inpatients”. Inpatients with previous contact in the primary care setting are referred to as “treatment-experienced inpatients”. The presence of complications upon hospitalization is confirmed by medical chart review of patients’ presenting symptoms and self-report. Expenditure data associated with the hospitalization are obtained from the claims data from the respective hospital’s financial and accounting offices. To measure patient severity at admission, we use patient vital signs and laboratory values for samples collected on the morning of the second day of admission, including height, weight, body mass index, blood pressure, blood sugar levels, liver and kidney functions, as well as other commonly collected indicators of diabetes progression such as neuropathy and retinopathy.

### Methods

To evaluate whether differences in the severity of illness exist between newly diagnosed inpatients and treatment-experienced inpatients, we report the means and, for continuous variables, standard deviations (SD) of socioeconomic and clinical variables for these two subgroups. Differences in means and their 95% confidence intervals are calculated. We also present proportions for categorical variables and their prevalence ratios, as well as the 95% confidence intervals around the ratios.

We conduct two separate multivariate regression analyses to examine our dual research questions. To investigate the differences in socioeconomic and clinical characteristics between newly diagnosed and treatment-experienced patients controlling for multiple covariates, we use multivariate probit regression with the dependent variable equal to 1 if the patient is newly diagnosed. Covariates in this multivariate probit regression include dummies for the years 2006 through 2008, for the type of insurance (full or partial insurance), female gender, area of residence (urban, county), employment and smoking status. HbA1c and patient age are the two continuous variables included in the regression. The omitted categorical variables are the year 2005, uninsured status, male gender, rural residence, full employment and non-smoking status. A second specification for this model replaces the continuous variable *hba* with a categorical variable set to 1 if *hba* > = 10%. Because we do not have random assignment into newly diagnosed and treatment-experienced groups, our empirical specification is primarily designed to characterize the differences between newly diagnosed and treatment-experienced patients conditional upon a hospitalization.

For the second research question, we use ordinary least squares regression to examine the correlations between HbA1c scores and the various socioeconomic variables of interest. We run two specifications of this regression: The first includes all covariates as listed in the previous paragraph (except HbA1c), and the second adds marital status, prior medication history (dummies for the categories of medication taken, i.e., oral medications or insulin), family history of diabetes, existence of complications upon admission, and duration of diabetes diagnosis. We report Huber-White heteroskedastic-robust standard errors.

The unbiasedness and efficiency of the probit and ordinary least squares models depends on the correct model specifications, and in particular omitted variable bias. We have included most of the traditional covariates in our data to examine the relationship between measures of diabetes severity and socioeconomic and clinical factors, but cannot exclude the possibility that omitted variables may be driving some of our findings. Because of limitations in the SRRSH data, our sample excludes readmission patients. For the SPH data, which include readmissions, we conduct analyses using not only new admissions but also the entire sample of observations to investigate the sensitivity of our results to different sample selection criteria.

This study has been approved by the Stanford University Institutional Review Board.

## Results

### Descriptive statistics

In the SRRSH sample, the average age was 56 years; 57% were male, 58% had less than a high school education, and 36% were uninsured. Patients in the SPH sample were slightly older, with an average age of 62, equally divided across genders, of which 21% were uninsured. We have no data on educational attainment for the SPH sample (Table 1).

In the SRRSH and SPH samples, respectively, 44% and 88% of patients were urban residents. Smokers accounted for 26% and 18% of the sample at SRRSH and SPH, respectively.

### Descriptive statistics: newly diagnosed vs. treatment-experienced inpatients

Descriptive statistics indicate that newly diagnosed patients in general have poorer clinical indicators of health than treatment-experienced inpatients. The newly diagnosed patients at SRRSH had a higher body mass index (BMI) (by 1.02 kg/m^2^, p < 0.01), higher fasting blood glucose (by 26.3 mg/dl, p < 0.01), and higher mean HbA1c (by 1.99 percentage points, p < 0.01). They also had other indicators of poorer health: higher cholesterol (by 12.73 mg/dl, p < 0.01), triglycerides (by 47.46 mg/dl, p < 0.05), LDL (by 6.47 mg/dl, p < 0.05), aspartate aminotransferase (AST) (by 11.45 IU/L, p < 0.01), and alanine aminotransferase (ALT) (by 12.01 IU/L, p < 0.01), but mixed indicators of renal function: creatinine (lower by 0.1 mg/dl, p < 0.1), blood urea nitrogen (BUN) (lower by 1.5 mg/dl, p < 0.1), and estimated glomerular filtration rate (eGFR) (higher by 11.22 ml/min/1.73 M^2^, p < 0.01).

Relative to treatment-experienced patients, newly diagnosed SPH patients also had higher BMI, fasting blood glucose, triglycerides, total cholesterol, and LDL, but these differences failed to achieve statistical significance at conventional levels. Newly diagnosed SPH patients did demonstrate, however, higher AST (by 6.8 IU/L, p < 0.1) and ALT (8.0 IU/L, p < 0.1).

Newly diagnosed patients were also more likely to report that they had complications when first diagnosed (e.g., 41% vs. 30% at SRRSH). At the time of discharge from SRRSH, 75% of the newly diagnosed patients were classified as having at least one chronic complication. The corresponding figure for SPH was 68%.

Overall, these statistics provide evidence that newly diagnosed patients are on average admitted for inpatient care with clinically worse indicators of health than treatment-experienced patients.

Because most of our regressions include multiple specifications, in the following section, we present the most conservative (smallest) result.

### Multivariate analysis: differences in socioeconomic and clinical characteristics of newly diagnosed and treatment-experienced patients

In Table 2, we report the conditional probabilities associated with various socioeconomic and clinical factors, given an inpatient admission for Type 2 DM, of being a newly diagnosed inpatient relative to a treatment-experienced inpatient. Again, given data limitations, results from Table 2 are meant only to highlight the socioeconomic and clinical differences between patients admitted virtually immediately upon diagnosis and patients who had preexisting contact with the primary care system for the treatment of diabetes. These results cannot predict prospectively that a patient with a given socioeconomic or clinical characteristic will become a newly diagnosed inpatient. Moreover, results could possibly be driven by sample selection given non-random assignment into the newly diagnosed and treatment-experienced groups, which we discuss further below.

**Table 2:** Differences in socioeconomic and clinical characteristics between newly diagnosed and treatment-experienced diabetic inpatients

	**Sir Run Run Shaw Hospital**		**Shandong Provincial Hospital**			
	**New admissions only†**		**All observations**		**New admissions only†**	
	**(1)**	**(2)**	**(3)**	**(4)**	**(5)**	**(6)**
**Dependent variable: first diagnosis of T2D within 6 months of inpatient admission (=1 if yes)**						
Patient age	-0.46	-0.39	-0.26	-0.26	-0.28	-0.28
	[-0.64 - -0.28)]	[-0.57 - -0.21]	[-0.38 - -0.14]	[-0.38 - -0.14]	[-0.42 - -0.15]	[-0.41 - -0.15]
*Female*	-6.37	-5.64	-2.54	-2.44	-2.66	-2.56
	[-11.2 - -1.52]	[-10.4 - -0.86]	[-6.23 - 1.15]	[-6.13 - 1.25]	[-6.80 - 1.48]	[-6.70 - 1.58]
*< High school*	6.02	5.41	Not available	Not available	Not available	Not available
	[1.59 - 10.4)]	[1.05 - 9.77]				
Insurance status						
*Insured*	1.99	2.53	-2.03	-2.15	-1.51	-1.52
	[-4.98 - 8.95]	[-4.51 - 9.58]	[-7.11 - 3.05]	[-7.15 - 2.85]	[-8.06 - 5.04]	[-8.07 - 5.03]
*Semi-insured*	-0.038	0.8	-6.80	-6.77	-7.00	-6.94
	[-4.89 - 4.82]	[-3.99 - 5.60]	[-12.8 - -0.84]	[-12.7 - -0.792]	[-13.4 - -0.61]	[-13.3 - -0.53]
Residence						
*Urban*	2.35	2.64	-0.578	-0.66	-0.97	-1.07
	[-3.00 - 7.71]	[-2.64 - 7.93]	[-5.75 - 4.60]	[-6.07 - 4.75]	[-6.93 - 4.99]	[-7.09 - 4.95]
*County/town*	-2.29	-0.71	Not available	Not available	Not available	Not available
	[-8.08 - 3.51]	[-6.74 - 5.31]				
*Smoking history*	-0.76	-0.46	-1.84	-1.73	-2.14	-2.02
	[-5.84 - 4.32]	[-5.50 - 4.59]	[-5.31 - 1.63]	[-5.24 - 1.78]	[-6.08 - 1.80]	[-6.00 - 1.96]
Serum glucose						
HbA1c %	1.95		0.56		0.60	
	[1.35 - 2.56]		[0.01 - 1.12]		[-0.024 - 1.23]	
*HbA1c% > 10%*		21.4		3.07		3.40
		[15.1 - 27.7]		[-0.144 - 6.28]		[-0.21 - 7.01]
Year fixed effects	Included	Included	Included	Included	Included	Included
Observations	900	900	822	822	741	741

All coefficients reported in % terms (decimal shifted two places to the right) to increase legibility.

95% confidence interval reported for marginal effects.

Year fixed effects are dummy variables for the years 2006, 2007, and 2008.

^†^“New admissions only” means that only the first inpatient observation of each unique patient ID is Included the sample.

Data: Inpatient admission for T2D, Sir Run Run Shaw Hospital (Hangzhou, China) and Shandong Provincial Hospital from 2005 to 2008. Patients under 18 are excluded.

Econometric model: Probit regression with dependent variable equal to 1 if patient admitted within 6 months of first diagnosis of T2D and 0 if admitted with a duration of diagnosis greater than 6 months. Categorical independent variables are italicized, so dy/dx represents the discrete change of dummy variables from 0 to 1.

Returning to the discussion of our results, we show that among patients admitted for hospitalization, multivariate probit regressions indicate that being younger, being male (for the SRRSH sample only), having less than a high school education (for the SRRSH sample only), and having high HbA1c levels were positively correlated with hospitalization as a newly diagnosed patient (Table 2) relative to treatment-experienced patients. In the SRRSH sample, conditional on an inpatient admission, each additional year in age is associated with a decrease in probability of 0.39% (p < 0.01, model (2)) for newly diagnosed hospitalization. Relative to treatment-experienced inpatients, newly diagnosed patients are also 5.64% (p < 0.01, model (2)) less likely to be female; and are 5.4% (p < 0.05, model (2)) more likely to be patients with less than a high school education. The association between lower educational attainment and being hospitalized upon diagnosis is evident in the SRRSH sample even when controlling for the higher HbA1c of such patients.

With respect to the association between lower education and newly diagnosed hospitalization, there is concern that the association is driven entirely by selection bias because of shorter survival rates associated with lower socioeconomic status, causing patients with lower education to be under-represented among the non-cases (treatment-experienced group). We address this concern below.

First, we note that in our full SRRSH sample, univariate analysis shows that patients with an education level of high school or lower represents 61% of newly diagnosed patients, and 57% of the treatment-experienced group. As indicated in Table 1, the null hypothesis of a unit prevalence ratio for lower than high school attainment between the two groups cannot be rejected.

Second, we restrict the data to patients admitted to the hospital within five years of initial diabetes diagnosis to reduce the difference in age of cases and non-cases, with an average patient age of 41.21 years (SD 6.77) for the newly diagnosed group, and 42.1 years (SD 5.56) for the treatment-experienced group. With this specification, the coefficient on the variable high school or less remains positive and significant, or 8.65% (p < 0.01).

Third, we further restricted the sample to patients less than fifty years of age and with a diabetes diagnosis of five years or fewer. In this sample, the newly diagnosed group had an average duration of diagnosis of 3.48 months (SD 7.32) and age of 42.21 years (SD 6.77) and the treatment-experienced group had an average duration of diagnosis of 25.45 months (SD 20.51) and age of 42.10 years (SD 5.56). In this probit regression, the coefficient on “high school or less” is 18.77% (p < 0.01).

Finally, we used the full sample and employed a nearest neighbor matching strategy, choosing the three observations from the non-cases (treatment-experienced patients) that are the closest match in gender, age, HbA1c scores, insurance status, residence type, and year of admission to each newly diagnosed inpatient. This specification also yields a statistically significant positive association (5.9%, p < 0.05) between lower education attainment and being a newly diagnosed inpatient. This estimate from the nearest neighbor matching method is remarkably close to the result from the OLS specification (5.4%, p < 0.05).

We now return to the results in Table 2. Among patients hospitalized for DM, each additional percentage point in HbA1c score is associated with a 1.95% (p < 0.01, model (1)) increase in the probability of a newly diagnosis hospitalization; and newly diagnosed inpatients are 21.4% (p < 0.01, model (2)) more likely to have an HbA1c score of 10% or more than treatment-experienced inpatients.

Turning to the results from the SPH data, we find again that among DM inpatients, each additional year of age is associated with a 0.26% (p < 0.01, models (3, 4)) decrease in the probability of newly diagnosed hospitalization. On the other hand, every percentage point increase in HbA1c score is positively associated (by 0.6%, p < 0.05, model (3)) with the likelihood of being a newly diagnosed rather than a treatment-experienced inpatient. Moreover, having HbA1c of greater than 10% is positively associated with being a newly diagnosed inpatient (3.1%, p < 0.1, model (4)). We had no data on patient educational attainment in the SPH sample.

### Association between HbA1c scores at admission and socioeconomic/clinical characteristics

In Table 3, the most consistent finding across samples is that patients with full insurance are less likely to be admitted for poorer serum glucose control among DM inpatients. In the SRRSH sample, fully insured patients have HbA1c scores that are 0.58 (p < 0.01, model (1)) lower. This correlation is also evident in the SPH data: insured patients have HbA1c percentages that are 1.62 (p < 0.01, model (4)) lower. The single socioeconomic factor that is associated with being admitted with lower levels of HbA1c is urban residence. In the SRRSH sample, we find that urban residents are admitted with HbA1c scores that are lower by 0.4 (p < 0.05, model (1)) percentage points. In addition, also in the more detailed SRRSH sample, prior medication history, which is itself likely to be highly correlated with past treatment experience in the primary care setting, is associated with lower HbA1c scores upon admission, by 1.26 (p < 0.01, model (2)) for patients on oral medication, and by 1.37 (p < 0.01, model (2)) for patients on insulin therapy.

**Table 3:** Factors associated with poor serum glucose control at time of hospital admission

	**Sir Run Run Shaw Hospital**		**Shandong Provincial Hospital**			
	**New admissions only†**		**New admissions only†**		**All admissions**	
**VARIABLES**	**(1)**	**(2)**	**(3)**	**(4)**	**(5)**	**(6)**
**Dependent variable: HbA1c**						
Demographic						
Patient age	-0.021	-0.019	-0.008	-0.0094	-0.011	-0.014
	[-0.034 - -0.0073]	[-0.033 - -0.0061]	[-0.024 - 0.0078]	[-0.026 - 0.0072]	[-0.026 - 0.0045]	[-0.03 - 0.002]
*Married*	Not included	0.23	Not included	-0.33	Not included	-0.49
		[-1.91 - -0.82]				
		[-0.49 - 0.95]		[-1.1 - 0.44]		[-1.19 - 0.22]
*Female*	-0.37	-0.28	0.21	0.19	0.16	0.13
	[-0.74 - 0.012]	[-0.66 - 0.095]	[-0.21 - 0.63]	[-0.23 - 0.62]	[-0.20 - 0.53]	[-0.24 - 0.50]
*Urban*	-0.37	-0.4	-0.47	-0.47	-0.39	-0.38
	[-0.73 - -0.018]	[-0.74 - -0.053]	[-1.23 - 0.30]	[-1.23 - 0.30]	[-1.14 - 0.35]	[-1.13 - 0.36]
*County/town*	-0.13	-0.082	Not available	Not available	Not available	Not available
	[-0.86 - 0.61]	[-0.83 - 0.67]				
*< High school*	-0.085	-0.14	Not available	Not available	Not available	Not available
	[-0.55 - 0.38]	[-0.59 - 0.32]				
*Unemployed*	-0.32	-0.28	0.011	0.014	0.063	0.064
	[-0.91 - 0.27]	[-0.86 - 0.30]	[-0.82 - 0.84]	[-0.82 - 0.85]	[-0.74 - 0.87]	[-0.74 - 0.87]
Past treatment						
*Oral agents*	Not included	-1.26	Not available	Not available	Not available	Not available
		[-1.71 - -0.81]				
*Insulin*	Not included	-1.37	Not available	Not available	Not available	Not available
		[-0.49 - 0.95]		[-1.1 - 0.44]		[-1.19 - 0.22]
Insurance						
*Insured*	-0.58	-0.59	-1.62	-1.62	-1.74	-1.74
	[-1.02 - -0.15]	[-1.01 - -0.17]	[-2.62 - -0.62]	[-2.62 - -0.62]	[-2.68 - -0.80]	[-2.68 - -0.80]
*Semi-insured*	-0.053	-0.0018	-0.41	-0.41	-0.45	-0.44
	[-0.42 - 0.31]	[-0.37 - 0.36]	[-1.20 - 0.37]	[-1.20 - 0.37]	[-1.21 - 0.32]	[-1.20 - 0.32]
*Clinical*						
*Smoker*	-0.083	-0.031	0.24	0.23	0.21	0.19
	[-0.44 - 0.29]	[-0.39 - 0.33]	[-0.22 - 0.69]	[-0.22 - 0.69]	[-0.26 - 0.68]	[-0.28 - 0.66]
Duration/months	0.0015	0.0039	-0.0017	-0.0017	-0.0016	-0.0015
	[-0.0072 - 0.010]	[-0.0048 - 0.013]	[-0.0041 - 0.00067]	[-0.0042 - 0.00070]	[-0.0038 - 0.00056]	[-0.0037 - 0.00069]
*Family history*	Not included	-0.33	Not included	-0.0036	Not included	-0.10
		[-0.74 - 0.078]		[-0.38 - 0.37]		[-0.45 - 0.24]
*Year fixed effects*	Included	Included	Included	Included	Included	Included
Observations	899	898	808	808	910	910
R-squared	0.04	0.08	0.05	0.05	0.06	0.06

95% confidence intervals in brackets. Coefficients are reported to two significant digits. Ordinary Least Squares Regression.

^†^“New admissions only” means that only the first inpatient observation of each unique patient ID is included the sample.

Data: Inpatient admission for T2D, Sir Run Run Shaw Hospital (Hangzhou, China) and Shandong Provincial Hospital (Jinan, China) from 2005 to 2008. Patients under 18 are excluded.

## Discussion

We find that diabetic patients hospitalized without prior primary care treatment experience or with poorer serum control differ from other diabetic inpatients along socioeconomic and clinical lines, and are more likely to be from traditionally vulnerable groups such as those with lower education attainment, without full insurance, or with rural residence. A significant minority of inpatients in our samples is hospitalized shortly after diagnosis, with no other treatment history for diabetes in the primary care setting. Our univariate analyses show that compared to treatment-experienced inpatients, newly diagnosed inpatients have worse indicators of health upon admission. Furthermore, our multivariate probit analysis, conditional on a diabetic inpatient admission, indicates that newly diagnosed patients tend to have low educational attainment in the SRRSH sample, even when controlling for HbA1c scores at admission. Second, high HbA1c levels are in turn negatively associated with full insurance status and, in the SRRSH sample, also negatively correlated with urban residence and preexisting access to oral medications and/or insulin.

These associations must be interpreted within the context of the available data for analysis. On the one hand, our finding of a socioeconomic and clinical gradient in avoidable hospitalizations is consistent with growing but sparse literature on health disparities in China among diabetic patients. Previous literature identified that lower education attainment is a risk factor for both pre-diabetes and diabetes in China [4,14], and our research provides preliminary evidence that these disparities may well extend into the inpatient setting, where the less educated are more likely to be admitted for hospitalizations with no prior primary care contact for the treatment of diabetes. On the other hand, our results may be driven by non-random assignment into the newly diagnosed and treatment-experienced groups. In particular, the association between lower education attainment and new diagnosis hospitalizations may be due to precocious deaths and underrepresentation of such patients in the treatment-experienced group. When we match the newly diagnosed and treatment experienced group on age and other observable covariates or restrict our analyses to younger patients (for whom precocious death is less of a concern), however, the association between lower education and arguably avoidable new diagnosis hospitalization remains.

Our study may also be the first to investigate the correlations between socioeconomic factors and poorer control of serum glucose levels (high HbA1c scores) at inpatient admission. We find that among patients hospitalized at tertiary hospitals in Eastern China, lack of or incomplete insurance coverage, and in the SRRSH sample, rural residence and drug therapy-naïve status, are correlated with higher HbA1c percentages. Taken together, our investigation of the two research questions demonstrates that lower educational attainment, rural residence, and/or lack of full insurance are associated with new diagnosis hospitalizations or greater severity of illness at admission for diabetic inpatients at two tertiary care centers in two coastal Chinese provinces. These findings point to potential signs of inequity in access to health care, and/or disparities in health behavior, literacy, and survival between patients of disparate socioeconomic status in China.

The association between lack of full insurance and hospitalization for diabetes in our study is consistent with the findings of a vast literature showing that financial constraints may delay needed medical care [17-20]. Individuals with less education may lack knowledge of diabetes and the roles of lifestyle, screening, symptoms, and good control in the course of the disease, as well as face more daunting access barriers to early and effective treatment. Although our study is observational in nature and limited to only two hospitals, it is consistent with a finding of socioeconomic gradients in health and in healthcare utilization. Such gradients may partly be related to health behaviors; better-educated Chinese even in rural areas are less likely to smoke, drink, or have a chronic disease [21]. Relative to the existing literature that focuses on diabetes prevalence or barriers to primary care, our study shows that such inequities may well be observable even at the *inpatient* level.

### Limitations

The principal limitation of our study is the lack of information on the hospitalization rates of diabetic patients treated as outpatients. As such, our results can best be interpreted as the likelihood of having certain socioeconomic and clinical factors conditional on inpatient admission for DM. Further, as noted previously, our finding of an association between lower education and new-diagnosis hospitalizations may have been driven by differential survival of such patients in our full sample. However, the association remains (and increases both in magnitude and precision) even when matching newly diagnosed and treatment-experienced patients on age, suggesting that sample selection is unlikely the sole reason for the education gradient found in our sample.

We also note the potential for recall bias in key variables in the study, including duration of diagnosis and complications at admission. However, due to the saliency of an inpatient admission soon after the first diagnosis of diabetes, it is unlikely that such a recall bias would be extensive. Moreover, complications present upon admission are recorded in medical charts and unlikely to be substantially affected by recall bias.

Finally, given the observational design, we are unable to uncover the underlying mechanism that links populations with lower socioeconomic status with a new-diagnosis hospitalizations or poorer serum glucose control. Financial constraints, lack of knowledge, preferences for large urban tertiary care institutions, or even spurious associations driven by sample selection would certainly require different policy responses to tackle the growing prevalence of chronic illnesses in China.

## Conclusion

Our findings of an association between greater severity of illness or hospitalizations upon first diagnosis and vulnerable groups such as individuals with lower education attainment, rural residence, and/or less than full insurance in two tertiary care hospitals are consistent with health disparities among diabetic patients in China. These inequalities may arise for a variety of reasons, including lack of access to primary care, health illiteracy, shorter survival, or other unobserved heterogeneity and selection in our study sample. Nevertheless, they point to a disturbing inequality in a country with an escalating diabetes prevalence rate. If these findings are driven by lack of access by vulnerable groups, China’s recent expansions of basic health insurance and investments in community health services represent an encouraging trend that may help to alleviate these disparities [22]. A salient goal of China’s recent health reforms is to increase use of primary care and decrease crowding at tertiary hospitals. An important marker of progress will be the extent to which primary care can prevent, detect, and control chronic diseases such as diabetes. As China continues on its health reform, future research should investigate whether these findings can be generalized to a larger population of Chinese diabetic inpatients, and whether the underlying cause of our observed disparities is driven by inequitable access to primary care along the urban/rural, insurance status, and education attainment divide.

## Abbreviations

ACSC: Ambulatory care sensitive conditions; CNY: Chinese Yuan; DM: Diabetes mellitus; SD: Standard deviation; SPH: Shandong Provincial Hospital; SRRSH: Sir Run Run Shaw Hospital (affiliated with Zhejiang University Medical School).

## Competing interests

The authors declare that they have no competing interests.

## Authors’ contributions

BC originated the research question for the study, conducted the analysis for the SRRSH data, and provided critical review and revision of the manuscript. KE wrote the initial manuscript based on the analysis performed by BC, and continued to provide critical review during subsequent revisions. NS provided critical comments on the analysis and manuscript. HL collected the SRRSH data and provided comments on the analysis and the manuscript. JW collected the SPH data and conducted the analysis thereon, as well as provided comments on the manuscript. All authors read and approved the final manuscript.

## Authors’ information

Brian Chen, JD, PhD

As an applied economist, Dr. Chen’s research focuses on the impact of incentives in health care organizations on provider and patient behavior. For his dissertation, Chen empirically examined how vertical integration and prohibition against self-referrals affected physician prescribing behavior. His job market paper was selected for presentation at the American Law and Economics Association’s Annual Meeting, the Academy of Management, the Canadian Law and Economics Association, the Conference on Empirical Legal Studies, and the First Annual Conference on Empirical Health Law and Policy at Georgetown Law Center in 2009. The paper was also nominated for best paper based on a dissertation at the Academy of Management.

Karen Eggleston, PhD

Karen Eggleston is director of the Walter S. Shorenstein Asia Pacific Research Center’s Asia Health Policy Program at Stanford University. She is also a fellow at Stanford’s Center for Health Policy/Primary Care and Outcomes Research (CHP/PCOR), and a Faculty Research Fellow of the National Bureau of Economic Research (NBER). Her research focuses on comparative healthcare systems and health reform in Asia, especially China; government and market roles in the health sector; payment incentives; healthcare productivity; and the economics of the demographic transition. Eggleston teaches through Stanford’s East Asian studies program and is also affiliated with Stanford’s public policy program.

Nilay Shah, PhD

Dr. Shah’s research is focused on studying and improving the health care delivery system. Dr. Shah has an ongoing research agenda for evaluating alternative models of chronic disease care delivery, medication adherence in chronic disease, policy implications of shared decision making and disparities in care. Other areas of ongoing research include optimizing treatment decisions in diabetes, decision analytic modeling of diagnostic strategies across a spectrum of diseases, evaluating the evidence base for quality measurement. Dr. Shah also has extensive experience working with large databases such as the Medical Expenditure Panel Survey (MEPS), National Ambulatory Medical Care Survey (NAMCS), and various payer and provider-based administrative data. Dr. Shah also has extensive experience with and continues to work on various topics related to pharmaceutical policy.

Hong Li, MD

Professor Li graduated from Zhejiang University School of Medicine (formerly Zhejiang Medical University) in 1982 and worked in the First Affiliated Hospital of Zhejiang University School of Medicine after graduation. She received her Master degree of Endocrine and Metabolic Diseases from Zhejiang University School of Medicine in 1989. She transferred to Sir Run Run Shaw Hospital, Zhejiang University School of Medicine in Nov. 2006. Li has received trainings at Harvard University Joslin Diabetes Center in the United States (Mar. 2003) and was a visiting scholar/visiting professor at Luebeck Medical University in Germany (1991–1992) as well as several universities in the United States, including Loma Linda University, Mayo Clinic, MD Anderson Cancer Center and Johns Hopkins University School of Medicine. Li was elected master instructor in 1996, and elected PhD supervisor in 2004. She has trained 40 graduate students and 6 Ph.D. students.

Jian Wang, PhD

Dr. Wang teaches at Shandong University’s Center for Health Management and Policy. He received his B.A. degree for Public Health from Shandong Medical University in 1993, a Master’s Degree for Health Statistics from Shandong Medical University in 1996, and a Doctoral Degree for Epidemiology from Shandong University from 2002. His research interests include Health Management, Health Policy, Health Economic and Social Medicine.
